# Potential therapeutic mechanisms of *Draconis Resina* in cardiovascular diseases-a narrative review

**DOI:** 10.3389/fphar.2025.1531873

**Published:** 2025-03-06

**Authors:** Jing Wang, Xiusong Tang, Peng Lan, Bin Liang, Yu Fang, Hongbo Li

**Affiliations:** ^1^ Department of Cardiology, Nanning Hospital of Traditional Chinese Medicine, Nanning, Guangxi, China; ^2^ Department of Science and Education, Nanning Hospital of Traditional Chinese Medicine, Nanning, Guangxi, China

**Keywords:** draconis resina, loureirin, cardiovascular diseases, pharmacology, cardioprotective effect

## Abstract

As a traditional Chinese herbal medicine, *Draconis Resina* (DR) has garnered significant attention due to its efficacy in treating various clinical diseases. Notably, it demonstrates remarkable therapeutic effects in cardiovascular diseases, such as atherosclerosis, coronary heart disease, and myocardial ischemia-reperfusion injury. A comprehensive understanding of the potential therapeutic mechanisms of DR in cardiovascular diseases can positively influence their prevention and treatment. Therefore, through a thorough literature review, this paper summarizes the primary mechanisms of DR in managing cardiovascular diseases, which include the prevention of thrombosis, inhibition of inflammatory responses, alleviation of oxidative stress, enhancement of endothelial function, and mitigation of myocardial fibrosis. There may remain many untapped therapeutic applications of DR that need further exploration. This review aims to give readers a deeper understanding of the DR and offer new perspectives.

## Graphical Abstract

**Figure:**
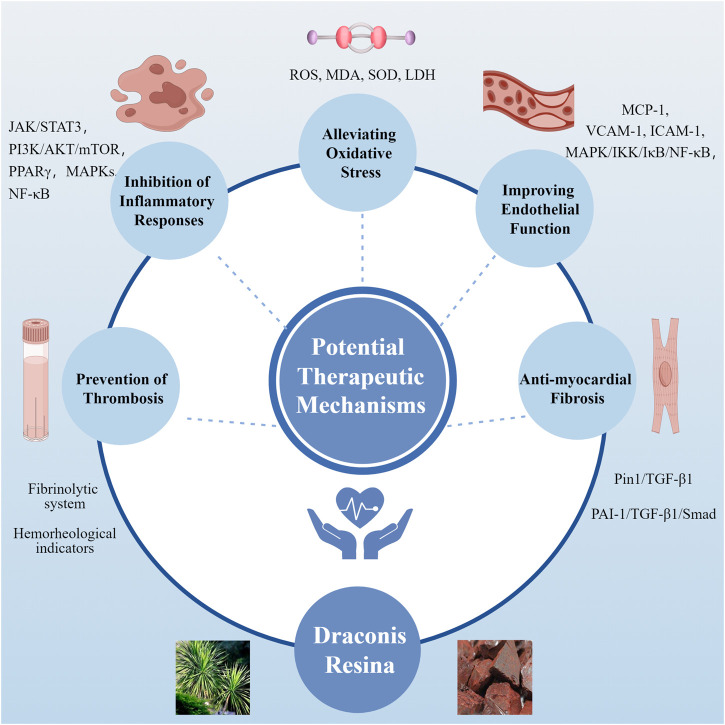
Mechanisms of *Draconis Resina*’s Protection Effects in Cardiovascular Diseases. (NF-κB: Nuclear factor-κB; ROS: Reactive oxygen species; MDA: Malondialdehyde; SOD: Superoxide dismutase; LDH: Lactate Dehydrogenase; MCP-1: Monocyte chemotactic protein - 1; ICAM-1: Intercellular adhesion molecule - 1; VCAM-1: Vascular cell adhesion molecule - 1).

## 1 Introduction

Cardiovascular disease, recognized as the leading cause of mortality threatening human health, is a focus of medical research (Zhao et al., 2023). In recent years, the distinctive advantages of traditional Chinese medicine in preventing and treating cardiovascular diseases have become increasingly evident, offering new hopes to overcome the critical health challenge. As a traditional Chinese medicine with a long history, *Draconis Resina* (DR), known in Chinese as Longxuejie, is derived from the *genus Dracaena* and is obtained through the ethanol extraction of its resinous wood (Wan et al., 2017). The *genus Dracaena* encompasses approximately 40 species of plants, among which *Dracaena cochinchinensis (Lour.) S.C.Chen* is the main source of DR. Globally, *Dracaena cochinchinensis (Lour.) S.C.Chen* is primarily distributed across Southeast Asian countries, including Cambodia, Laos, and Vietnam. In China, it is mainly distributed in the tropical and subtropical limestone mountains with an altitude ranging from 250 to 1700 m in southern Guangxi and southern Yunnan. DR has attracted much attention due to thousands of years of records demonstrating its effectiveness in promoting blood circulation, alleviating blood stasis, reducing swelling, and relieving pain. It has been widely used in the treatment of various injuries and illnesses in traditional practices. With the rapid development of modern science and technology, researchers have begun to investigate the medicinal mechanisms of DR, particularly its potential value in cardiovascular diseases (Fan et al., 2014). Pharmacological studies have gradually unveiled its multifaceted effects on the cardiovascular system. Currently, some research teams are dedicated to identifying the active ingredients of DR and analyzing their targeted signaling pathways. They hope it will integrate ancient medicine into the clinical treatment of cardiovascular diseases accurately, thereby providing patients with more effective treatment strategies and alleviating the burden of cardiovascular conditions. With the continuous deepening of research on cardiovascular diseases, it is believed that the potential of DR will play a more prominent role in the field of medicine in the future. This review aims to provide a comprehensive perspective on the potential mechanisms of DR in cardiovascular diseases.

## 2 Extraction technique of *Draconis Resina*

In pharmacological studies, the main chemical components of DR are the focus of research. Obtaining extracts from the original medicine of DR is necessary for pharmacological studies. The extraction technologies for DR primarily include traditional alcohol extraction and non-heating extraction methods. The alcohol extraction method utilizes ethanol and other organic solvents to extract the active components through processes, such as heating and reflux. The resulting extract is then filtered, concentrated, and dried to yield the DR extract (Li et al., 2014). Although this process is well-established, it is associated with high energy consumption and may have limitations in extraction efficiency. Some studies also mentioned that using ethyl acetate as an extraction solvent can significantly improve the extraction efficiency of DR (Tu et al., 2011; Wang et al., 2015). The non-heating extraction method employs innovative techniques, such as the pressure cross-variation method, which applies pressure to the solvent that has been absorbed by the medicinal materials. This pressure alters the geometric structure of plant cells, enhancing the osmotic pressure across the cell wall and thereby facilitating the dissolution of the cellular components. Following solid-liquid separation and drying using a low-temperature air heat balance method, the extract is obtained (Hu et al., 2002). This process could achieve high extraction efficiency, ensure complete extraction of components, and result in a loosely structured extract that enhances absorption and therapeutic efficacy but does not require an external heat source. Additionally, this method conserves plant resources, reduces the quantity of raw materials needed, and lowers treatment costs. With the development of various technologies, some other extraction technologies are also gradually emerging, such as ultrasonic-assisted semi-bionic extraction technology. Research shows that the ultrasonic-assisted semi-bionic extraction technology is simple, stable and feasible, and can be used for the extraction of DR (Gao et al., 2021). The conventional purification process of DR use macroporous resin for purification. This method effectively increases the concentration of total flavonoids and other active compounds in DR, while simultaneously removing a majority of impurities and significantly improving product quality (Zhou et al., 2019).

## 3 Chemical composition of *Draconis Resina*

The main chemical constituents of DR include phenols, terpenoids, steroids, *etc.* Among them, phenolic compounds are the characteristic of its main active ingredients (Peres et al., 2023). To date, researchers have isolated and identified over 180 types of phenolic compounds in plants of the *genus Dracaena* (Sun et al., 2019). Among these components, flavonoids and stilbenes, which serve as the foundational structures, are relatively abundant and exhibit medicinal properties. Researchers have summarized and analyzed the literature related to DR over the past decade. The findings indicate that more than 80 types of flavonoids have been extracted, isolated, and identified from DR (Lin et al., 2020), encompassing categories such as dihydrochalcone compounds (including Loureirin A, Loureirin B, and Loureirin C), chalcones, flavans, dihydroflavonoids, and flavones. Among these, Loureirin A and Loureirin B are the primary active ingredients, so they are frequently employed in the quality control and pharmacodynamic research of DR (Zhang et al., 2019). By searching the literature (Wang X. et al., 2024; Lin et al., 2020), we summarized more than 100 chemical components of DR (Table 1).

**TABLE 1:** Chemical components of *Draconis Resina*.

Compounds	Category
Loureirin A	Dihydrochalcones
Loureirin B	Dihydrochalcones
Loureirin C	Dihydrochalcones
Loureirin D	Dihydrochalcones
Cambodianin A	Dihydrochalcones
Cambodianin B	Dihydrochalcones
Cochinchinenin A	Dihydrochalcones
Cochinchinenin B	Dihydrochalcones
Cochinchinenin D	Dihydrochalcones
Cochinchinenin I	Dihydrochalcones
2,3′-Dihydroxy-4,4′-dimethoxydihydrochalcone	Dihydrochalcones
2,4,4′-Trihydroxy-3′-methoxy-3-methyldihydrochalcone	Dihydrochalcones
2,4,4′-Trihydroxy-3-methyldihydrochalcone	Dihydrochalcones
2,4′-Dihydroxy-4,6-dimethoxydihydrochalcone	Dihydrochalcones
2,4′-Dihydroxy-4-methoxydihydrochalcone	Dihydrochalcones
2,4-Dimethoxy-6,4′-dihydroxydihydrochalcone	Dihydrochalcones
4,4′-Dihydroxy-2,3′-dimethoxydihydrochalcone	Dihydrochalcones
4,4′-Dihydroxy-2,6-dimethoxydihydrochalcone	Dihydrochalcones
4,4-Dihydroxy-2,6-dimethoxydihydrochalcone	Dihydrochalcones
4-Hydroxy-2′,4′-dimethoxydihydrochalcone	Dihydrochalcones
2′,4′,4-Trihydroxy-3-methoxychalcone	Chalcones
2′,4,4-Trihydroxychalcone	Chalcones
3,2′,3′,4′-Tetrahydroxy-4-methoxychalcone	Chalcones
3,2′,4′-Trihydroxy-4-methoxychalcone	Chalcones
3-Deoxysappanchalcone	Chalcones
4,2′,4′-Trihydroxychalcone	Chalcones
4,4′-Dihydroxy-2′-methoxychalcone	Chalcones
Sappanchalcone	Chalcones
(±)-7,4′-Dihydroxy - 3′-methoxyflavone	Flavonoids
(±)-7,4′-dihydroxy - dihydrohomoisoflavone	Flavonoids
(3R)-7,4′-dihydroxy - 6 - methoxyhomoisoflavone	Flavonoids
(3S)-7,3′-dihydroxy - 4′-methoxydihydrohomoisoflavone	Flavonoids
(7R,12bR)-7,10 - Dihydroxy - 4,11 - dimethoxydracorhodin	Flavonoids
(7S,12BS)-10,11 - Dihydroxy - 1 - methoxydracorhodin	Flavonoids
(7S,12BS)-10,11 - Dihydroxy - 1 - methoxydracorhodin	Flavonoids
(7S,12BS)-11 - Hydroxy - 1,10 - dimethoxydracorhodin	Flavonoids
10 - Hydroxy - 11 - methoxydracorhodin	Flavonoids
10,11 - Dihydroxydracorhodin	Flavonoids
4′,5,7 - Trihydroxyflavone	Flavonoids
4,5,7 - Trihydroxyisoflavone	Flavonoids
4,7 - Dihydroxy - 8 - methylisoflavone	Flavonoids
4,7 - Dihydroxyisoflavone	Flavonoids
4′-Dihydroxy - 5 - methoxydihydroisoflavone	Flavonoids
5,7,4′-Trihydroxydihydroflavone	Flavonoids
7 - Hydroxydihydroflavone	Flavonoids
7 - Hydroxyflavone	Flavonoids
7,4′-Dihydroxy - 8 - methylflavone	Flavonoids
7,4′-Dihydroxydihydroflavone	Flavonoids
7,4′-Dihydroxydihydroflavone	Flavonoids
7,4′-Dihydroxydihydroisoflavone	Flavonoids
7,4′-Dihydroxyflavone	Flavonoids
7,4′-Dihydroxyflavone	Flavonoids
7,4′-Dihydroxyflavone	Flavonoids
7,4′-Dihydroxyisoflavane	Flavonoids
Kumatakenin B	Flavonoids
(2S)-7,3′,4′-Trihydroxy - 8 - methylflavan	Flavans
(2S)-7,3′-Dihydroxy-4′-methoxyflavan	Flavans
(2S)-7,4′-Dihydroxy-8-methylflavan	Flavans
4,7 - Dihydroxy - 3 - methoxyflavan	Flavans
4′,7 - Dihydroxy - 3′-methoxy - 8 - methylflavan	Flavans
4′,7 - Dihydroxyflavan	Flavans
4′-Hydroxy - 7 - methoxy - 8 - methylflavan	Flavans
4′-Hydroxy-3′,7-dimethoxyflavan	Flavans
4′-Hydroxy-7-methoxy-8-methylflavan	Flavans
4′-Hydroxy-7-methoxy-8-methylflavan	Flavans
5,4′-Dihydroxy - 7 - methoxy - 6 - methylflavan	Flavans
5,4′-Dihydroxy - 7 - methoxy - 6 - methylflavan	Flavans
5,7-Dihydroxy-4′-methoxy-8-methylflavan	Flavans
6,4′-Dihydroxy-7-methoxyisoflavane	Flavans
7 - Hydroxy - 4′-methoxyflavan	Flavans
7 - Hydroxy - 8 - methyl - 4′-methoxyflavan	Flavans
7,3′-Dihydroxy - 4′-methoxyflavan	Flavans
7,4′-Dihydroxy - 8 - methylflavan	Flavans
7,4′-Dihydroxy-5-methoxy-8-methylflavan	Flavans
7,4′-Dihydroxy-8-methoxyisoflavane	Flavans
7,4′-Dihydroxy-8-methylflavan	Flavans
7,4′-Dihydroxyflavan	Flavans
7,4′-Dihydroxyflavan	Flavans
7,4′-Dihydroxyisoflavane	Flavans
7-Hydroxy-4′-methoxy-8-methylflavan	Flavans
7-Hydroxy-4′-methoxyflavan	Flavans
Dracophane	Dihydrochalcane trimers
Nordracophane	Dihydrochalcane trimers
Epipinoresinol	Lignans
Medioresinol	Lignans
Pinoresinol	Lignans
Syringaresin	Lignans
Syringaresinol	Lignans
3,4′-Dihydroxy - 5 - methoxystilbene	Stilbenes
3,5 - Dihydroxystilbene	Stilbenes
3,5 - Dimethoxystilbene	Stilbenes
3,5,4′-Trihydroxystilbene	Stilbenes
4 - Hydroxy - 3,5 - dimethoxydistyryl	Stilbenes
4′-Hydroxy - 3,5 - dimethoxystilbene	Stilbenes
Pterostilbene	Stilbenes
(1β,3β,14α,20R,22S,25R) - Spirost - 5 - ene - 1,3,14 - triol	Saponins and Sterols
(20S,22R,25R) - Spirost - 1α,3β - dihydroxy - 5 - ene	Saponins and Sterols
(25R) - Diosgenin	Saponins and Sterols
(25R) - Spirost - 5 - en - 3β - ol	Saponins and Sterols
(25R) - Spirost - 5 - ene - 3β,17α - diol	Saponins and Sterols
(25S) - Spirost - 5 - en - 1β,3β,14α - triol 22S - Spirostanesteroids	Saponins and Sterols
4 - Methyl - cholest - 7 - en - 3β - ol	Saponins and Sterols
4α - Methylcholest - 7 - en - 3β - ol	Saponins and Sterols
Campesterol	Saponins and Sterols
Daucosterol	Saponins and Sterols
Stigmasterol	Saponins and Sterols
β - Sitosterol	Saponins and Sterols
1 - Hydroxy - 6,8 - dimethoxy - 3 - methylanthraquinone	Others
2,4 - Dihydroxyacetophenone	Others
3 - Methylresveratrol	Others
3,4 - Dihydroxyallylbenzene	Others
3,4,5 - Trimethoxyphenol	Others
Balanophonin	Others
Cochinchinenin C	Others
Cochinchinenin J	Others
Coumarin	Others
Protocatechualdehyde	Others
Resveratrol	Others
Trans - 4′,5 - Dihydroxy - 3 - methoxystilbene	Others

## 4 Pharmacokinetics and bioavailability of *Draconis Resina*

At present, few studies on the pharmacokinetics and bioavailability of DR. Only a few studies have explored the relevant characteristics of DR extract, such as Loureirin A. Following oral administration in rats, Loureirin A is distributed through the blood circulation to various tissues. The liver and kidney had the highest concentration, followed by the lungs, spleen, heart and brain, while demonstrating limited distribution in peripheral tissues (Sun et al., 2018). One study examined the binding rate of Loureirin A to human plasma proteins, revealing that there is a strong affinity between them. It may influence Loureirin A’s free concentration and bioavailability *in vivo* (Sun, 2009). However, further research is necessary to elucidate the absorption process of Loureirin A in the body. Limited data are currently available regarding the metabolism and excretion processes of Loureirin A *in vivo*. Generally, drugs and their metabolites are primarily excreted via urine and feces. Li et al. identified the metabolic characteristics of five flavonoids in DR (Loureirin A, Loureirin B, Loureirin C, 7,4'-dihydroxyflavanones, and 5,7,4'-trihydroxyflavanones) in human liver microsomes. A total of 29 metabolites of the above five compounds were identified, and hydroxylation, oxidation and demethylation were found as the primary biotransformation pathways (Li et al., 2017). Zhang et al. used the HPLC-MS/MS method to determine the Loureirin A and Loureirin B in rat urine, feces, and bile after oral administration of 10.6 g/kg of DR. Calibration curves of loureirin A in rat urine, feces, and bile were linear over the concentration range of 1–5,000 ng/mL. Loureirin B in rat urine, feces, and bile ranged between 0.08 and 20, 0.2 and 20, and 0.1 and 500 ng/mL, respectively (Zhang et al., 2011). Pharmacokinetic and tissue distribution observations were conducted in rats after a single 500 mg/kg oral dose. Rapid absorption (Tmax, 11.53–68.27 min) and elimination (T_1/2_, 6.893–57.90 min) occurred for all analytes of interest. Extensive occurrences were observed for 7,4'- dihydroxy - 5 - methoxyhomoisoflavanone (Cmax, 340.0 ng/mL), thevetiaflavone (Cmax, 42.86 ng/mL), 5,7,4'- trihydroxyhomoisoflavanone (Cmax, 41.55 ng/mL), and pterostilbene (Cmax, 25.49 ng/mL) in plasma. Significant distributions occurred for all analytes in the liver as well as the kidney, and several compounds could be found in the brain (Sun et al., 2018). Sun Xiaobo used HPLC to determine the pharmacokinetic parameters of Loureirin A in rats and beagle dogs. The dosage was set as 2.5 g/kg for rats and 250 mg/kg for dogs. The results suggested that the Tmax respectively were 2.06 h and 3 h (Sun, 2009). The pharmacokinetics of cochinchinenin A, 3,4′- dihydroxy - 5 - methoxystilbene, Loureirin A, and Loureirin B after oral dosage of DR were determined by other researchers. The results indicated that these phenolic compounds could be rapidly absorbed (Tmax lower than 2 h) (Ma et al., 2010; Zhao and Chen, 2013). Li et al. developed a novel method for the quantification of loureirin B in rat plasma using HPLC-MS/MS. The validated method was successfully applied to a preliminary pharmacokinetic study of loureirin B in rats (Li et al., 2009). After oral administration of 16 g/kg DR to rats, the main pharmacokinetic parameters Tmax, Cmax, T_1/2_, Ke and AUC_0-T_ were 0.8 h, 7.99 μg/L, 1.941 h, 0.365/h, and 22.21 μ·h/L, respectively. In addition, the comparative pharmacokinetic study in rats showed Loureirin A, Loureirin C and 7,4-dihydroxyflavone had decreased Cmax and AUC and increased Vd and CL in a simulated microgravity environment; but pterostilbene had the opposite changes. The four phenolic components also showed increased or decreased excretions in simulated microgravity rats (Li et al., 2016). The pharmacokinetic characteristic of DR is complex. Although some *in vivo* pharmacokinetic studies have been conducted on active ingredients in DR, such as Loureirin A, Loureirin B and Loureirin C, additional research is essential to fully understand the pharmacokinetic properties of DR as a whole. Such studies will enhance our understanding of the efficacy and mechanisms of action of DR, providing a scientific basis for its rational clinical application.

## 5 The application of *Draconis Resina* in cardiovascular diseases

### 5.1 Atherosclerosis

The stenosis and even occlusion of coronary arteries caused by atherosclerosis is the recognized pathological basis of coronary atherosclerotic heart disease (CHD). The prevention and treatment of atherosclerosis are of positive significance for reducing the incidence of CHD. Longxue Tongluo Capsule (LTC) is a novel drug composed of total phenolic extracts from DR. In an atherosclerotic model of ApoE-deficient mice induced by a high-fat diet (HFD), administration of low, medium, and high doses of LTC resulted in significant reductions in the atherosclerotic lesion area of the aortic sinus by 26.4% (p < 0.05), 30.1% (p < 0.05), and 46.5% (p < 0.01), respectively (Zheng et al., 2016a). However, Compared to ezetimibe, LTC did not demonstrate a significant effect on the improvement of blood lipid levels in this study. In hypercholesterolemic rats induced by a HFD, LTC exhibited a significant impact on regulating blood lipid levels. It notably reduce serum total cholesterol, triglycerides, and low-density lipoprotein cholesterol, while simultaneously increase high-density lipoprotein cholesterol levels (p < 0.001) (Zhou et al., 2018). In addition, the pathological examination of aortic tissue showed that LTC reduced the accumulation of atherosclerotic lesions due to mild diffuse thickening and mild multifocal fragmentation of elastic and collagen fibers in media. The varying effects of LTC on blood lipid levels observed in the two models may be attributed to factors, including the inherent differences between the models, the complexity of the drug’s pharmacological effects, and the species-specific responses to LTC. Future research should investigate these discrepancies to enhance our understanding of LTC’s mechanisms. Additionally, regarding the specific mechanisms by which LTC reduces atherosclerotic lesions, particularly its influence on elastic and collagen fibers, further studies are necessary to elucidate the underlying mechanisms and signaling pathways.

**FIGURE 1:**
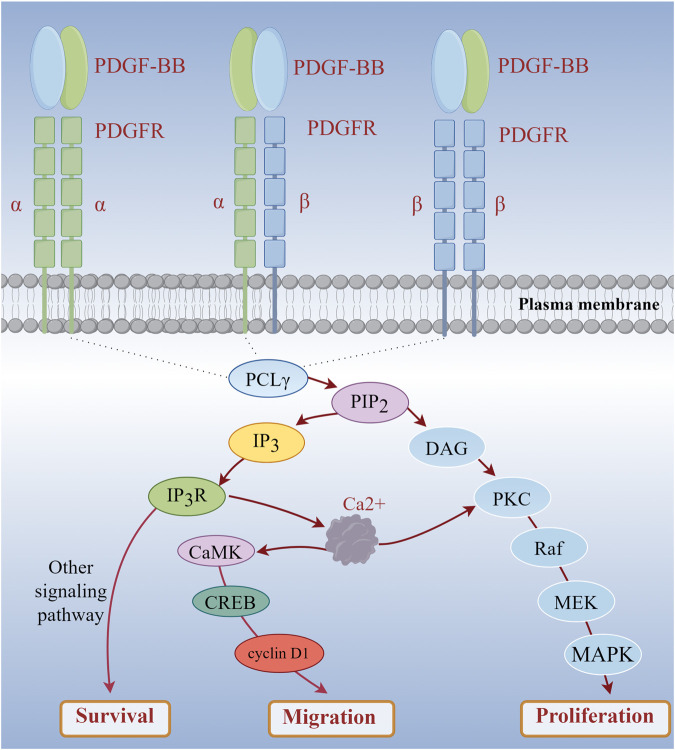
PDGF-BB Mediates the Increase of Intracellular Calcium Level to Induce Cell Proliferation and Migration. (PDGF-BB: platelet-derived growth factor-BB; PDGFR: platelet-derived growth factor receptor).

Vascular smooth muscle cell migration and proliferation are key processes in the neointima formation that occurs during atherosclerosis, and these events can be induced by platelet-derived growth factor (PDGF) (Won et al., 2011). Research has demonstrated that 2 μg/mL of LTC can significantly inhibit the migration of A7r5 cells induced by PDGF-BB (20 ng/mL) (p < 0.05) and markedly reduce the accumulation of Ca^2+^ in these cells (Zhou et al., 2018). When PDGF-BB binds to cell surface receptors, the phospholipase C-γ signaling pathway is activated. Phospholipase C-γ can catalyze the hydrolysis of phosphatidylinositol - 4, 5 - bisphosphate into inositol - 1, 4, 5 - trisphosphate and diacylglycerol. Inositol - 1, 4, 5 - trisphosphate subsequently interacts with its receptor on the endoplasmic reticulum, leading to the release of calcium from intracellular stores and a consequent increase in cytosolic Ca^2+^ concentration (Woll and Van Petegem, 2022; Huang et al., 2024). The elevated intracellular Ca^2+^ levels can activate calcium/calmodulin-dependent protein kinase (CaMK) (Najar et al., 2021). CaMK phosphorylates various transcription factors, including CREB (cAMP response element-binding protein), thereby promoting the expression of cell cycle-related genes such as cyclin D1, which facilitates cell cycle progression from G0/G1 to S phase (Liu et al., 2013). When A7r5 cells were treated with a calcium chelating to reduce intracellular Ca^2+^ levels, it was observed that PDGF-BB-induced cell proliferation was significantly inhibited. This finding indicates that increased intracellular Ca^2+^ levels are essential for the PDGF-BB-induced proliferation of A7r5 cells (Figure 1). In addition, during cell migration, cytoskeletal rearrangement plays a crucial role. Elevated intracellular Ca^2+^ levels triggered by PDGF-BB stimulation can activate small GTPases, particularly members of the Rho family (Vazão et al., 2011). These proteins can regulate the polymerization and depolymerization, altering cell morphology and motility.

Based on these findings, it is hypothesized that LTC may disrupt PDGF-BB signal transduction by lowering intracellular Ca^2+^ levels. This disruption in signal transduction ultimately inhibits the proliferation of A7r5 cells induced by PDGF-BB (20 ng/mL). MAPKs is an important pathway involved in cell proliferation, which may be related to the proliferation of A7r5 cells induced by PDGF-BB (Figure 1). However, the current understanding of these mechanisms is incomplete. More detailed and specific studies are necessary to elucidate how LTC regulates the signaling pathways associated with intracellular Ca^2+^ levels and to determine whether other targets or pathways are involved in the regulation of cell proliferation and migration. This understanding is essential for a comprehensive grasp of the role of LTC in the prevention and treatment of atherosclerosis.

### 5.2 Coronary heart disease

CHD represents a significant global public health challenge. The exploration of new therapies is crucial for its prevention and treatment. DR, the primary component of Compound Longxuejie Capsule (CLC), has been the focus of many studies aimed at evaluating its efficacy in treating coronary heart disease.

**FIGURE 2:**
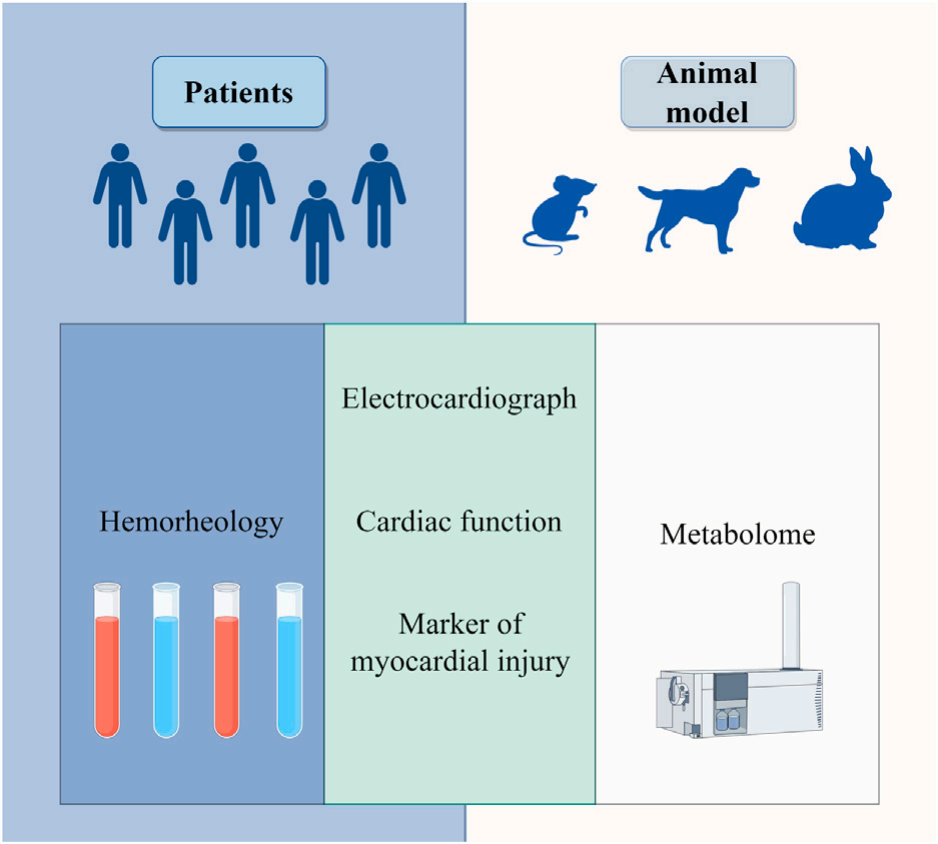
Clinical efficacy of *draconis resina* in the treatment of coronary heart disease in hemorheology, electrocardiogram, cardiac function, markers of myocardial injury and metabolome.

In the treatment of patients with acute myocardial infarction, the CLC, when used as an adjunct to conventional Western medicine, can reduce the left ventricular end-diastolic diameter, improve the levels of myocardial injury markers (Bao et al., 2015), enhance the overall clinical treatment effect (Li et al., 2014), and improve blood viscosity in the patient’s hemorheology (Wang, 2003). In the animal experiment, total flavonoids from DR can counteract the changes observed in the J point and T wave of the electrocardiogram in rats with myocardial ischemia. Additionally, it can reduce the infarcted myocardial area and the incidence of ST segment elevations in dogs subjected to coronary artery ligation, while also lowering relevant serum indicators such as creatine kinase (CK), lactate dehydrogenase (LDH), and lactate (Fang et al., 2005). In rabbits with acute myocardial infarction, DR demonstrates a protective effect on left heart function, mitigates the disorder of myocardial tissue structure, slows the heart rate, and stabilizes heart function indicators. It includes preventing significant decreases in left ventricular systolic pressure, the maximum rate of rise of left ventricular pressure (+dp/dtmax), and the maximum rate of fall of left ventricular pressure (-dp/dtmax), as well as avoiding a significant increase in left ventricular end-diastolic pressure (Cheng et al., 2017a). When DR was used to intervene in the mouse model of myocardial infarction, metabolomic analysis revealed significant alterations in the levels of seven myocardial infarction metabolic markers: phytosphingosine, sphinganine, acetylcarnitine, cGMP, cAMP, L-tyrosine, and L-valine (Qi et al., 2013). These findings suggest that DR may improve outcomes in myocardial infarction by regulating vascular smooth muscle contraction, sphingolipid metabolism, phenylalanine metabolism, and branched-chain amino acid metabolism (Figure 2). Su Xin investigated the mechanism of the CLC in a rat model of myocardial infarction using network pharmacology and quantitative real - time polymerase chain reaction (Su X. et al., 2024). The results indicated that the PI3K/Akt pathway plays a crucial role in the anti-myocardial ischemia effects of the CLC. In patients with stable coronary heart disease, the CLC demonstrated superior efficacy in alleviating angina pectoris and improving electrocardiogram outcomes compared to the control group (Qing et al., 2009), while also enhancing the patients’ hemorheological status (Jiang et al., 2015). A randomized, double-blind, parallel controlled, multi-center clinical trial involving 418 patients showed that CLC is effective and safe in the treatment of stable angina pectoris (Qing et al., 2009).

It is important to note that the CLC is a compound formulation comprising multiple ingredients. Apart from DR, it includes various other traditional Chinese medicine components or excipients, such as panax notoginseng and borneol. The presence of these additional ingredients may influence the research outcomes. From the perspective of traditional Chinese medicine, DR and panax notoginseng are both blood circulation-activating drugs. Research showed that panax notoginseng protected human umbilical vein endothelial cells from H_2_O_2_-induced oxidative stress. The mechanism is inhibiting early cell apoptosis via upregulating vascular endothelial growth factor A (VEGFA) mRNA expression (Dong et al., 2019). In addition, panax notoginseng could improve angiogenesis in rats with myocardial infarction and hypoxic human coronary artery endothelial cells, also affect the level of miR200a promoter methylation and miR200a and VEGF molecular pathways (Wang J. et al., 2024). For borneol, its cardiovascular benefits include reducing atherosclerosis by inhibiting foam cell formation through its metabolites (He et al., 2024). These therapeutic effects are of critical importance in the treatment of CHD. Consequently, it should be recognized that the effects observed from the CLC in studies are not entirely representative of the effects attributed to DR. To mitigate the potential interference of other components on research findings, studies investigating the effects of DR should ideally utilize it in isolation to enhance the accuracy and reliability of the results. Furthermore, while current research indicates certain therapeutic benefits, there remains a need for more extensive, multi-center clinical trials to further validate its safety and efficacy.

### 5.3 Myocardial ischemia-reperfusion injury

The mechanisms underlying myocardial ischemia-reperfusion injury (MIRI) are complex and involve a variety of physiological and pathological processes. These processes include oxidative stress, disruptions in energy metabolism, myocardial cell apoptosis, calcium overload, autophagy, cell infiltration, and vascular endothelial dysfunction. These pathological mechanisms can lead to significant damage to myocardial cells.

Research has demonstrated that total flavonoids from DR can reduce the concentration of LDH in the culture medium of a hypoxic/reoxygenated neonatal rat cardiomyocyte model, thereby enhancing cell viability (Deng et al., 2006). In animal experiments, these flavonoids exhibit a protective effect in rats subjected to MIRI, as they can decrease the area of myocardial infarction and the apoptosis rate of cardiomyocytes, while also reducing serum levels of CK, creatine kinase MB (CK-MB), and LDH to some extent (Liang et al., 2017). Similar findings were observed in experiments involving New Zealand rabbits with MIRI. Total flavonoids from DR were shown to improve myocardial tissue condition, stabilize the structure of myocardial cell membranes, reduce the leakage of myocardial CK-MB, and enhance myocardial systolic function. CK, CK-MB, and LDH serve as markers for cardiomyocyte necrosis (Cheng et al., 2017b). During MIRI, the release of these markers in blood correlates positively with the extent of myocardial necrosis and reflects the severity of MIRI damage. Collectively, these findings indicated that total flavonoids from DR could lower serum levels of CK, CK-MB, and LDH in animal models of MIRI, demonstrating a significant protective effect on cardiomyocytes in MIRI animals. Further research suggested that this protective effect may be closely associated with the JAK2/STAT3 signaling pathway (Cheng, 2017).

High mobility group box 1 protein (HMGB1) serves as an ancillary transcription factor within the cell nucleus, interacting with DNA and various transcription factors to regulate gene transcription. When cells are damaged or stimulated, HMGB1 can be released extracellularly, functioning as a significant inflammatory mediator. Extracellular HMGB1 can activate diverse immune cells, including macrophages and neutrophils, prompting them to release inflammatory factors and subsequently initiate an inflammatory response. In the myocardial cells of the ischemia/reperfusion (I/R) rat model, irregular cell arrangement is observed, with the intercellular matrix exhibiting signs of edema and some cells displaying necrosis. Additionally, infiltration of eosinophils and neutrophils is evident. Pretreatment of the I/R rat model with total flavonoids from DR could ameliorate these conditions and significantly reduce the expression of HMGB1 and PI3K proteins in myocardial tissue (Su L. et al., 2024). So, the mechanism through which the total flavonoids from DR alleviate MIRI may be associated with the antagonism of the HMGB1/PI3K signaling pathway and the subsequent reduction of the inflammatory response.

Apoptosis of cells plays a crucial role in the mechanism of ischemia-reperfusion injury. The PI3K/AKT signaling pathway regulates various cellular activities, including apoptosis. Caspase-3 protein is a key executor of cell apoptosis. Experimental evidence demonstrated that the activation of the PI3K/AKT signaling pathway could reduce the expression of caspase-3 protein (Zhou et al., 2023). Meanwhile, the expression of anti-apoptotic proteins such as Bcl-2 and Bcl-xL was promoted and the activity of the pro-apoptotic protein Bax was inhibited. This suggested that the activation of the PI3K/AKT pathway may inhibit the apoptosis of myocardial cells during MIRI, thereby exerting a protective effect. In tree shrews with DR reperfusion after myocardial ischemia, it also has been observed that the expression of Bcl-2 protein in myocardial tissue while the downregulating of Bax protein levels. Concurrently, the expressions of GRP78, CHOP-1, p-P38, p-JNK-1, and p-ERK proteins were also inhibited (Yang et al., 2021). GRP78 serves as a representative protein of endoplasmic reticulum stress, while CHOP-1 is a key transcription factor involved in endoplasmic reticulum stress-induced apoptosis. The proteins p-P38, p-JNK-1, and p-ERK belong to the MAPK family and play significant roles in the regulation of apoptosis. Therefore, reperfusion of the myocardium with DR can reduce cell apoptosis, inhibit endoplasmic reticulum stress to a certain extent, and ultimately achieve a protective effect against MIRI. Further research showed that DR inhibited endoplasmic reticulum-induced apoptosis of myocardial cells may by regulating the miR-423-3p/ERK signaling pathway in the tree shrew myocardial I/R model (Yang et al., 2019).

Pyroptosis is a newly defined programmed cell death mode characterized by the formation of pores in the cell membrane, leading to an imbalance in intracellular and extracellular osmotic pressure and ultimately resulting in cell lysis. The process releases pro-inflammatory cytokines such as IL-1β and IL-18, which contribute to inflammatory response. The Caspase-3/GSDME pathway is one of the molecular mechanisms underlying pyroptosis. When activated, this signaling pathway induces pyroptosis in cardiomyocytes (Zheng et al., 2020). The study has shown that total flavonoids from DR can reduce serum levels of CK-MB and LDH in I/R rats, while also exerting a protective effect on myocardial cells subjected to I/R injury (Huang et al., 2022). Specifically, these flavonoids mitigate the loss of myocardial cell nucleus, decrease the area of myocardial infarction, reduce the degree of myocardial tissue disarray, and limit the infiltration of inflammatory cells in the myocardial interstitial. Notably, these protective effects coincide with a reduction in the expression levels of caspase-3, GSDME, and IL-1β mRNA. It suggests that inhibition of the Caspase-3/GSDME signaling pathway can partially counteract the pyroptosis induced by MIRI.

Some researchers have observed in the MIRI rat model that the total flavonoids from DR can upregulate the expression of Wnt2 protein, increase serum VEGF levels, and downregulate β-catenin protein expression (Liang et al., 2023). Wnt2 primarily functions to promote cell proliferation, while β-catenin serves as a transcription factor that regulates downstream genes associated with apoptosis. This suggests that the total flavonoids from DR may enhance Wnt2 expression and negatively regulate β-catenin expression, thereby promoting angiogenesis and mitigating myocardial cell damage resulting from MIRI.

**FIGURE 3:**
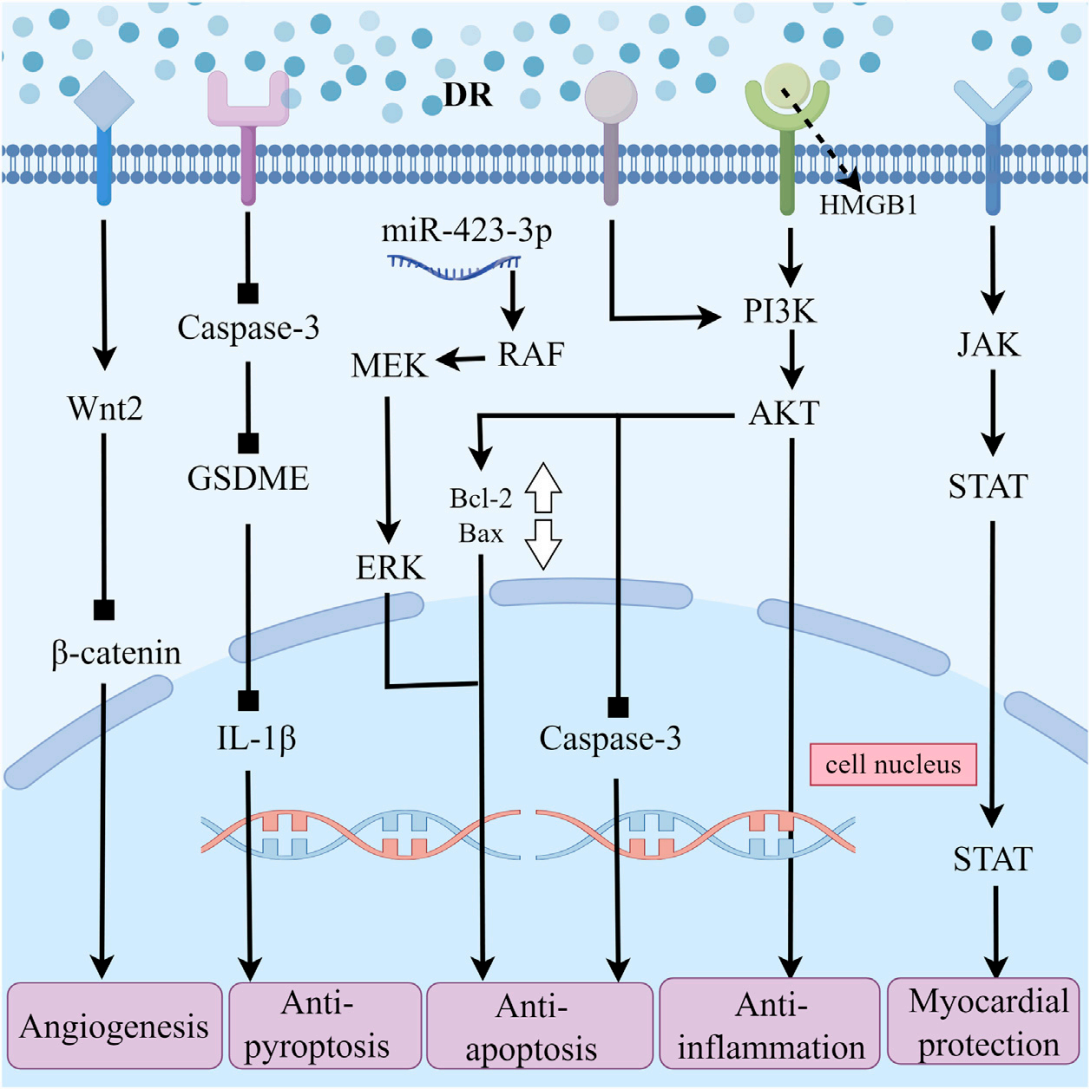
The Multiple Pathways of *Draconis Resina* Treating MIRI. (DR: *Draconis Resina*; MIRI: Myocardial ischemia-reperfusion injury).

In summary, DR can improve cardiac function-related indicators, such as CK, CK-MB, and LDH, in various animal species MIRI models. The primary protective effects of DR include the inhibition of inflammation, apoptosis, cell pyroptosis, and the promotion of angiogenesis. The mechanisms involved are linked to the JAK2/STAT3, HMGB1/PI3K, PI3K/AKT, Caspase-3/GSDME, and Wnt2/β-catenin signaling pathways. DR consistently demonstrates protective effects against MIRI in different animal models and methodologies, highlighting its therapeutic value (Figure 3).

## 6 Mechanisms of the *Draconis Resina* on cardiovascular diseases

### 6.1 Prevention of thrombosis

Increased fibrinogen (Jay et al., 1991), erythrocyte aggregation (Schechner et al., 2005), whole blood viscosity (Kanakaraj and Singh, 1989) plasma viscosity (Leonhardt et al., 1977), decreased osmotic fragility and deformability (Kanakaraj and Singh, 1989) are hemorheological factors associated with cardiovascular disease. These factors can significantly increase the risk of thrombosis, promote the occurrence of cardiovascular disease, and aggravate the progression. It's been suggested in the literature that DR exhibited clinical antithrombosis efficacy similar to low molecular weight heparin (Liang et al., 2021). Zheng Jiao found that treating hyperlipidemia mice with LTC caused beneficial changes in hemorheology, including a reduction in whole blood viscosity. Still, the deformation curve integral area of the erythrocyte also increased and the improvement of osmotic fragility (Zheng et al., 2016b).

**FIGURE 4:**
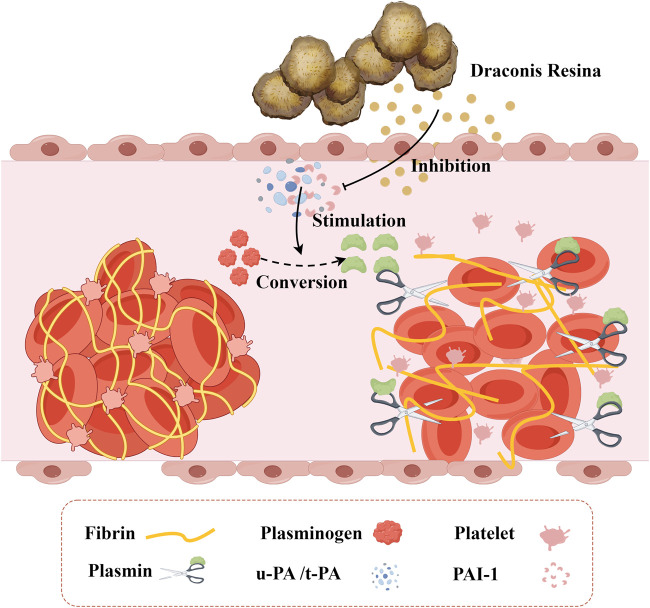
The Inhibitory Effect of *Draconis Resina* on Thrombosis. *Draconis Resina* inhibits PAI-1 and reduces the binding of PAI-1 to uPA/t-PA, which promotes the conversion of plasminogen into plasmin, thus decreasing the formation of thrombus. (u-PA: Urokinase-type plasminogen activatora; t-PA: Tissue plasminogen activator, PAI-1: Plasminogen activator inhibitor-1).

**FIGURE 5:**
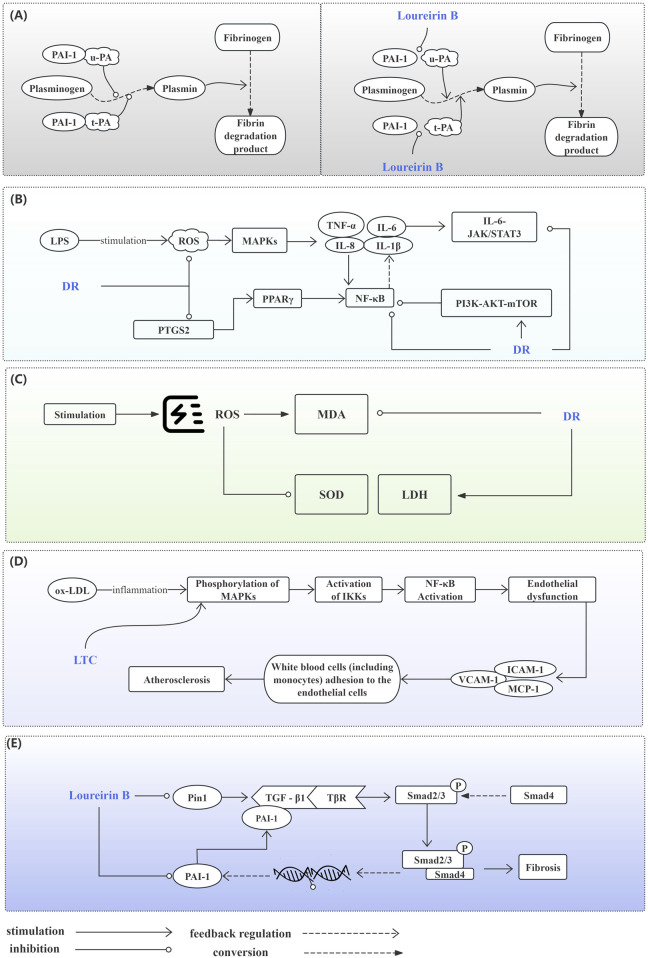
Potential Mechanisms of the *Draconis Resina* on Cardiovascular Diseases. **(A)** Prevention of Thrombosis. Plasmin participates in the conversion of fibrinogen to fibrin degradation product. Normally, u-PA and t-PA could promote plasminogen conversion to plasmin. PAI-1 inhibits plasminogen conversion when combined with u-PA or t-PA (left). Loureirin B can reduce the binding of PAI-1 to u-PA or t-PA and promote Plasminogen conversion (right). **(B)** Inhibition of Inflammatory Responses. DR alleviates inflammation by regulating MAPKs, PPAR, NF-κB, JAK/STAT3, PI3K/AKT/mTOR signaling pathways. **(C)** Alleviating Oxidative Stress. DR improves oxidative stress by affecting MDA, SOD and LDH levels. **(D)** Improving Endothelial Function. LTC regulates the MAPK/IKK/IκB/NF-κB signaling pathway to improve endothelial function. **(E)** Anti-myocardial Fibrosis. DR regulates the TGF-β1/Smad signaling pathway affected by PIN1 and PAI-1 against fibrosis. (AKT: Protein kinase B; DR: *Draconis Resina*; ICAM-1: Intercellular adhesion molecule - 1; IKK: IκB kinase; IL-1β: Interleukin - 1β; IL-6: Interleukin - 6; IL-8: Interleukin - 8; JAK: Janus kinase; LDH: Lactate dehydrogenase; LPS: Lipopolysaccharide; LTC: Longxue tongluo capsule; MAPK: Mitogen - activated protein kinase; MCP-1: Monocyte chemotactic protein - 1; MDA: Malondialdehyde; mTOR: Mammalian target of rapamycin; NF-κB: Nuclear factor - κB; ox-LDL: Oxidized low - density lipoprotein; PAI-1: Plasminogen activator inhibitor - 1; PI3K: Phosphoinositide 3 - kinase; Pin1: Peptidyl - prolyl cis - trans isomerase NIMA - interacting protein 1; PPAR: Peroxisome proliferator - activated receptor; PTGS: Prostaglandin - endoperoxide synthase; ROS: Reactive oxygen species; SATAT: Signal transducer and activator of transcription; SOD: Superoxide dismutase; TGF-β1: Transforming growth factor - β1; TNFα: Tumor necrosis factor - α; t-PA: Tissue - type plasminogen activator; TβR: Transforming growth factor - β receptor; u-PA: Urokinase - type plasminogen activator; VCAM-1: Vascular cell adhesion molecule - 1).

When blood vessels are damaged, tissue plasminogen activator (t-PA) can rapidly bind to specific regions of fibrin via its lysine residues. This binding subsequently activates plasminogen that is closely associated with fibrin, converting it into active plasmin. Plasmin, a potent hydrolase, can cleave polymerized fibrin within thrombi and transform it into soluble, low-molecular-weight products. The process could lead to the disruption and dissolution of the thrombus structure. Similarly, urokinase-type plasminogen activator (u-PA) exerts antithrombotic effects by promoting plasminogen activation, facilitating thrombolysis, and enhancing microcirculation through analogous mechanisms. Plasminogen activator inhibitor-1 (PAI-1) is a secreted single-chain glycoprotein and a member of the serine protease inhibitor family. In the fibrinolytic system, PAI-1 serves as the natural and specific inhibitor of both uPA and tPA. Abnormal expression of PAI-1 can disrupt the body’s coagulation and anticoagulation systems, potentially leading to thrombotic diseases and an increased risk of cardiovascular events. Therefore, inhibiting the abnormal expression of PAI-1 contributes to reducing the formation of thrombotic events. Loureirin B is an important flavonoid compound extracted from DR. Based on the plasma fibrin experiment, researchers employed the substrate chromogenic method to assess the inhibitory effect and dose dependence of Loureirin B on PAI-1, in addition to conducting a plasma fibrin experiment (Yan, 2016). The results indicated that Loureirin B effectively inhibits PAI-1, prevents the formation of the complex between PAI-1 and uPA, and exhibits a dose-dependent effect (Figure 4). Findings from the plasma fibrin experiment revealed that the activity of PAI-1 is inversely proportional to the concentration of Loureirin B inhibitor, whereas the activity of uPA is directly proportional. Consequently, the rate of fibrin dissolution in blood increases, demonstrating a significant antithrombotic effect. Figure 5A.

### 6.2 Inhibition of inflammatory responses

**FIGURE 6:**
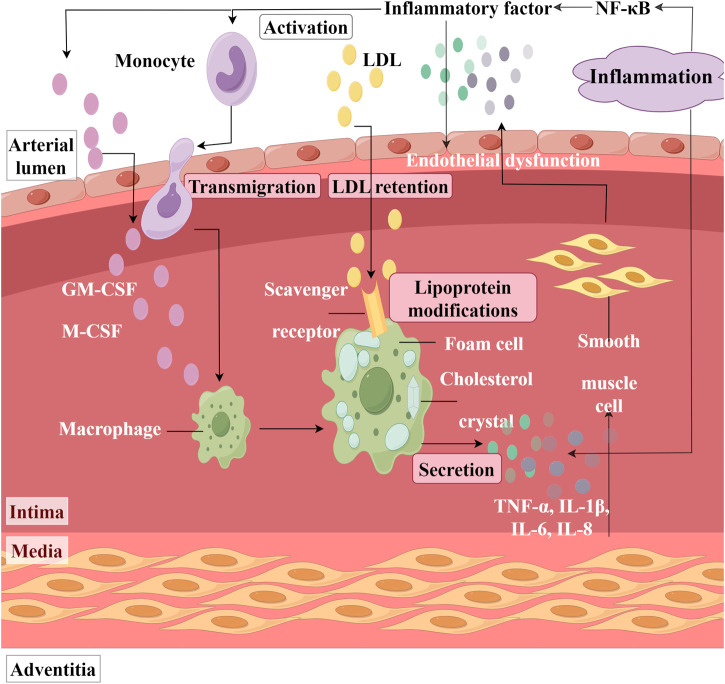
Inflammation in the Artery. (NF-κB: Nuclear factor kappa B, LDL: Low-density lipoprotein; GM-CSF: Granulocyte macrophage colony stimulating factor, M-CSF: Macrophage colony stimulating factor, IL: Interleukin, TNF-α: Tumor necrosis factor-alpha).

Nuclear factor kappa B (NF-κB) is a significant transcription factor that plays a crucial role in various physiological and pathological processes. In the inflammatory response, NF-κB serves as one of the primary regulatory factors. It can induce the expression of genes encoding various inflammatory mediators, such as TNF-α, IL-1β, IL-6, and IL-8, thereby facilitating the recruitment and activation of inflammatory cells and the releasing of inflammatory mediators (Figure 6). Research indicates that LTC treatment can decrease the number of NF-κB-positive stained microvessels in the aortic tissue of atherosclerotic rats. It suggests that LTC exerts an inhibitory effect on the activation and expression of NF-κB (Zhou et al., 2018). Additionally, Huang Biaohua and colleagues have demonstrated that the total flavonoids from DR can reduce the serum levels of inflammatory factors TNF-α and IL-6 in rats suffering from MIRI. Quantitative real - time polymerase chain reaction analysis reveals that the mRNA expression levels of TNF-α and IL-6 are downregulated (Huang et al., 2021).

It was reported that lipopolysaccharide (LPS)-induced human aortic smooth muscle cells (HASMC) proliferation and inflammation lead to the development of atherosclerosis (Yang et al., 2005). HASMC and macrophages activated by LPS secrete various inflammatory mediators, including TNF-α, IL-1β, IL-6, and IL-8 (Liu et al., 2007; Heo et al., 2008). Sook-Kyoung Heo found that the ethylacetate extract from DR can inhibit the production of TNF-α, IL-8, and IL-6 in LPS-treated HASMC and RAW264.7 macrophages (Heo et al., 2010). This inhibition is accompanied by a decrease in reactive oxygen species (ROS) levels. Notably, when RAW264.7 cells were pretreated with the NADPH oxidase assembly inhibitor AEBSF to inhibit ROS production before LPS stimulation, it was observed that the activation of MAPKs was suppressed, resulting in a simultaneous reduction of inflammatory factor levels. This suggested that the mechanism by which ethyl acetate extract from DR decreases inflammatory factor levels is associated with ROS-mediated activation of MAPKs.

Zhu Qianwei concluded through network pharmacology and molecular docking methods that the active ingredients in the CLC bind to targets such as AKT1 and PTGS2, influencing inflammatory signaling pathways (NF-κB and PPAR), thereby contributing to the treatment of CHD (Zhu et al., 2023). Researchers have identified that disorders in the IL-6-JAK/STAT3 pathway and the PI3K-AKT-mTOR pathway may represent significant pathological mechanisms underlying acute myocardial infarction. DR demonstrates a synergistic cardioprotective effect by regulating these two pathways and the expression of targets (VEGF, COX2, and PPARγ) (Li et al., 2018). Figure 5B.

### 6.3 Alleviating Oxidative Stress

**FIGURE 7:**
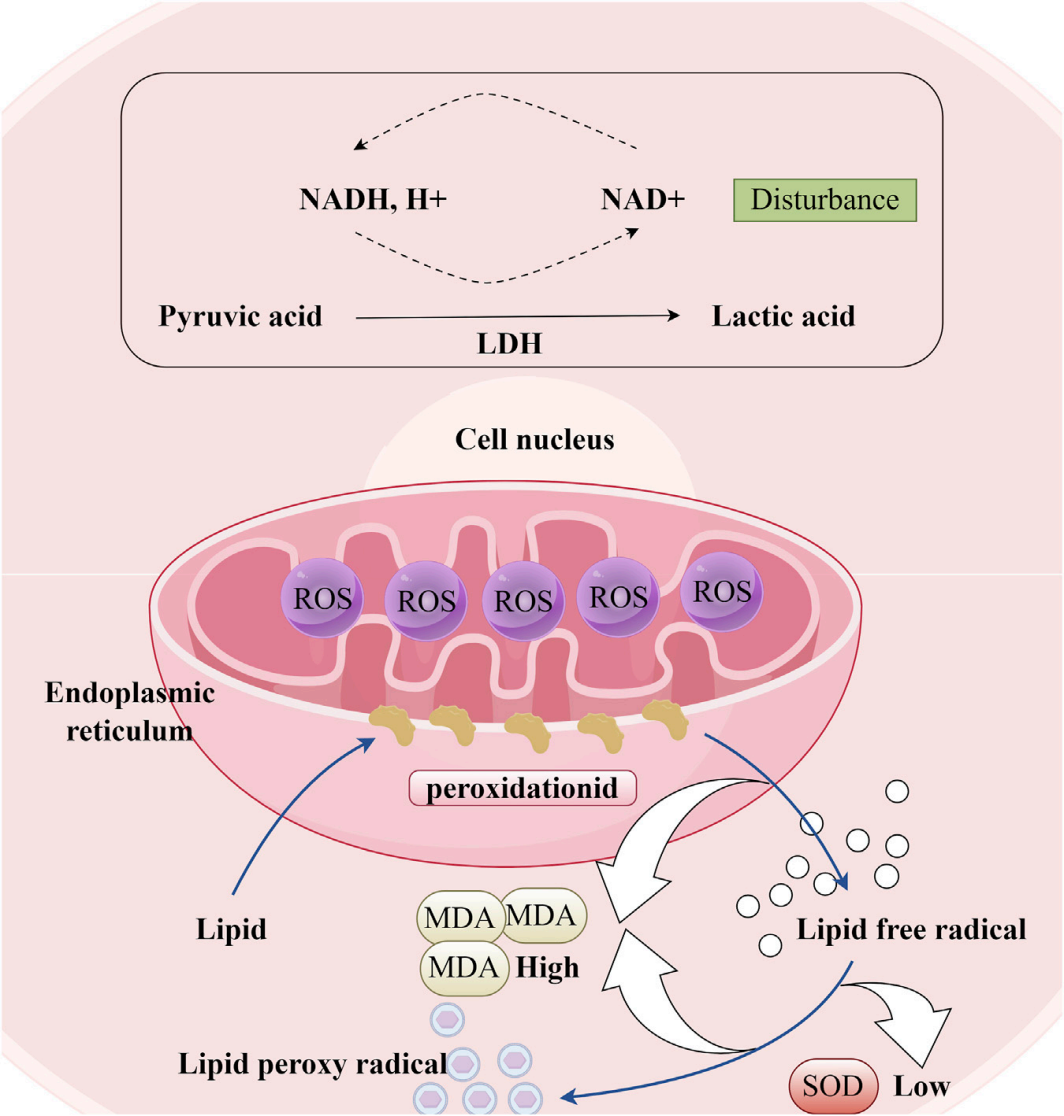
Oxidative Stress Response in Cardiomyocytes. (LDH: Lactate dehydrogenase, ROS: Reactive oxygen species; MDA: Malondialdehyde, SOD: Superoxide dismutase).

Lipid peroxidation, which refers to the oxidative degradation of lipids resulting in cell membrane damage, is considered a predictive biomarker for atherosclerosis and cardiovascular diseases (Rao et al., 2011). Superoxide dismutase (SOD) and malondialdehyde (MDA) are both biomarkers closely associated with oxidative stress. MDA is primarily one of the final products of lipid peroxidation reactions in living organisms and a direct indicator of myocardial lipid peroxidation damage (Figure 7). It has been reported that lipid accumulation and I/R can cause cellular lipid peroxidation damage (Wang et al., 2009; Han et al., 2018). LTC has been shown to reduce MDA levels in the serum of hyperlipidemic ApoE^−/−^ mouse models subjected to a high-fat diet (Zheng et al., 2016b). In their research, Yang Tianrui and colleagues discovered that, in addition to decreasing MDA levels in the myocardial tissue of tree shrews with I/R injury, DR also significantly promotes an increase in SOD (Yang et al., 2018). However, the role of these biomarkers in cellular redox balance and their impact on the function and survival of cardiomyocytes has not been thoroughly investigated. Therefore, future studies must elucidate the mechanisms by which DR alleviates oxidative stress to achieve cardiovascular protection, which is significant for the development of its potential pharmacological effects.

LDH is a glycolytic enzyme that is widely present in human cells (Figure 7). Under conditions of oxidative stress, the intracellular redox balance becomes disrupted, leading to the attack of excessive free radicals on the active center, amino acid residues, and coenzyme binding sites of LDH. This disruption results in altered LDH activity. The total flavonoids from DR have been shown to reduce LDH concentrations in the culture medium of a neonatal rat cardiomyocyte hypoxia/reoxygenation model, while also enhancing the viability of cardiomyocytes damaged by Na_2_S_2_O_4_ stimulation (Deng et al., 2006). Typically, mild oxidative stress may induce a compensatory increase in LDH activity to satisfy the energy metabolism demands of cells under stress. However, as oxidative stress intensifies, LDH activity may be inhibited due to excessive oxidative damage that compromises the protein structure and function of LDH. The observation that LDH concentration decreased rather than increased in the aforementioned study suggests that the oxidative stress experienced by cardiomyocytes stimulated by Na2S2O4 is severe. Figure 5C.

### 6.4 Improving Endothelial Function

**FIGURE 8:**
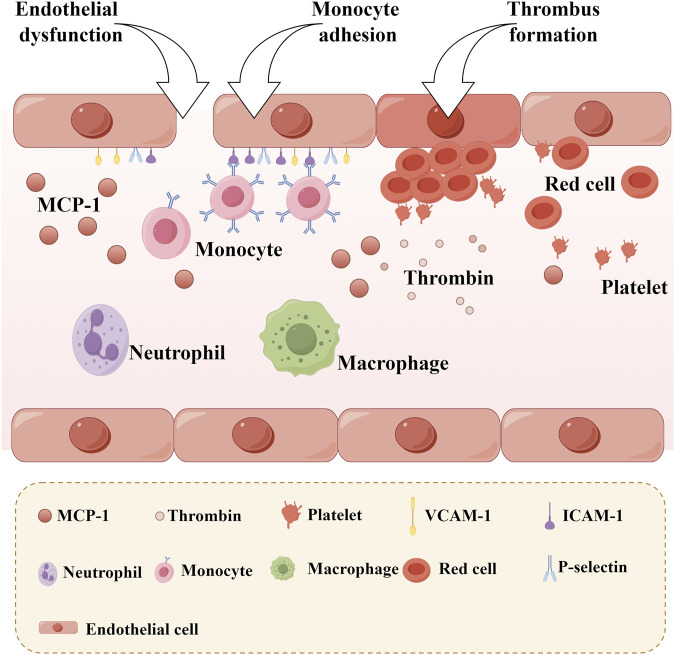
Monocyte Adhesion and Thrombosis after Endothelial Dysfunction. (MCP-1: Monocyte chemotactic protein-1, VCAM-1: Vascular cell adhesion molecule-1; ICAM-1: Intercellular adhesion molecule-1).

The increased adhesiveness of endothelial cells, resulting from impaired endothelial function, is the initial step in the formation of atherosclerosis. Zheng Jiao stimulated human umbilical vein endothelial cells with oxidized low-density lipoprotein and observed that the adhesion of these cells to monocytes significantly increased. Notably, LTC can significantly reverse this phenomenon, which is closely related to the regulation of the MAPK/IKK/IκB/NF-κB signaling pathway (Zheng et al., 2016a). Endothelial cells are primary producers of adhesion molecules, such as vascular cell adhesion molecule-1 (VCAM-1) and intercellular adhesion molecule-1 (ICAM-1), and they secrete chemotactic factors like monocyte chemotactic protein-1 (MCP-1) (Figure 8). These molecules play pivotal roles in the progression of atherosclerosis. Mediated by ICAM-1 and VCAM-1, the adherence of white blood cells to vascular endothelial cells directly impairs endothelial integrity or induces endothelial dysfunction. This process facilitates the migration of white blood cells toward the vascular endothelium and stimulates the proliferation of vascular smooth muscle cells. Concurrently, the emergence of MCP-1 attracts monocytes within the bloodstream, causing them to adhere to the vascular endothelium and migrate beneath it. These monocytes differentiate into macrophages, engulfing cholesterol to form foam cells. Ultimately, this sequence of events leads to the development of atherosclerosis. The study found that a 4-week LTC intervention can reduce the serum levels of VCAM-1, ICAM-1, and MCP-1 in atherosclerotic model rats (Zhou et al., 2018). This result suggests that LTC may influence the expression of ICAM-1, VCAM-1, and MCP-1 through specific potential action pathways (such as the NF-κB signaling pathway), thereby playing a beneficial role in the prevention and treatment of atherosclerosis. These findings provide a vital research direction for further exploration of the specific mechanisms by which LTC may prevent and treat atherosclerosis. Figure 5D.

### 6.5 Anti-myocardial fibrosis

**FIGURE 9:**
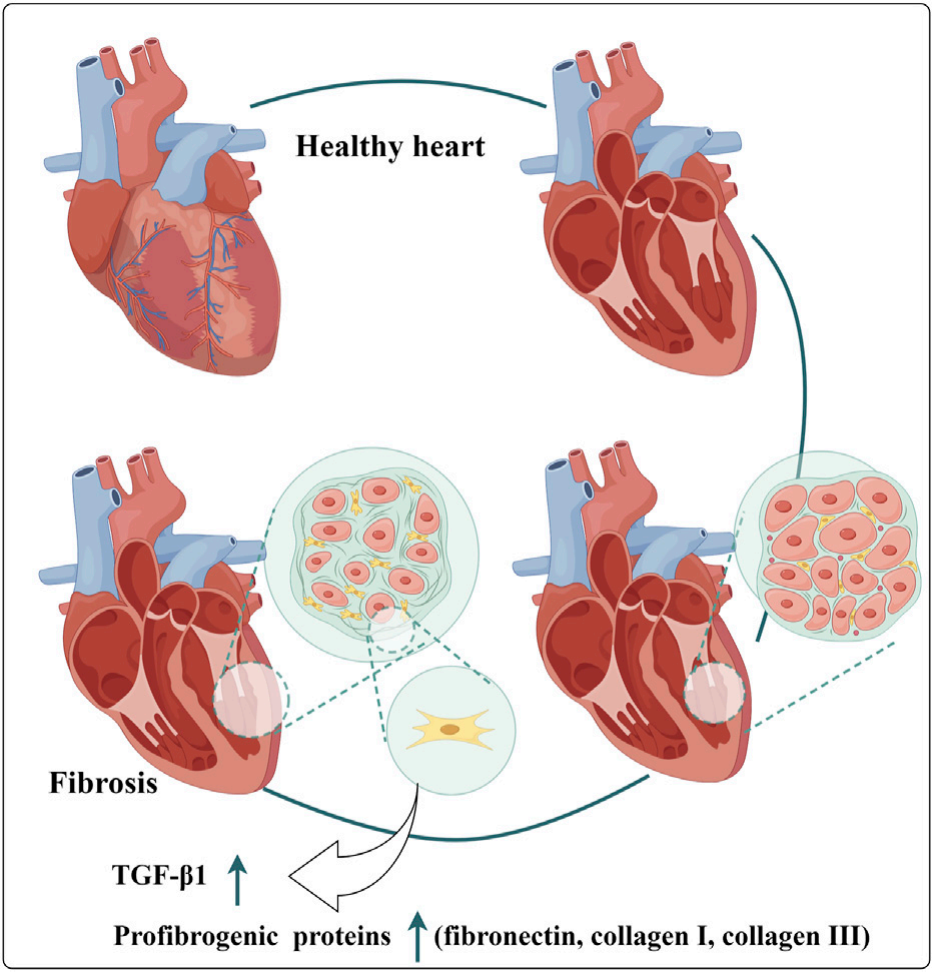
Elevated Myocardial Fibrosis Related Indicators. (TGF-β1: transforming growth factor-β1).

In the MIRI model, Western blot analysis demonstrated a significant increase in the expression of fibrosis-related proteins, including fibronectin, collagen I, collagen III, and α-SMA, in the cardiac tissue of MIRI mice (Figure 9). Following a 4-week intervention with Loureirin B, the elevated levels of these proteins were effectively reduced, indicating that Loureirin B treatment can significantly mitigate collagen fiber deposition induced by MIRI (Kong and Zhao, 2022). Particularly, Loureirin B also inhibited the expression of pro-fibrotic factors such as PAI-1, transforming growth factor-β1 (TGF-β1), TGF-β1 receptor, and phosphorylated Smad2/3. TGF-β1 is known to play a crucial role in the occurrence and progression of myocardial fibrosis, while Smad proteins serve as key downstream effectors in the TGF-β1 signaling pathway. Increasing evidence suggests that PAI-1 is involved in the pathogenesis of various fibrosis-related diseases, including liver, lung, and kidney fibrosis (Oda et al., 2001; Wang et al., 2007; Courey et al., 2011). Thus, inhibiting the PAI-1/TGF-β1/Smad signaling pathway may provide a promising strategy for alleviating myocardial fibrosis induced by MIRI (Kong and Zhao, 2022). Additionally, Pin1 has been implicated in fibrotic diseases, including cardiac fibrosis, and can modulate the production of TGF-β1 (Wu et al., 2017). The study found that Loureirin B can also ameliorate cardiac fibrosis both *in vitro* and *in vivo* via the Pin1/TGF-β1 signaling pathway (Cheng et al., 2022). Figure 5E.

## 7 Outlook

DR is composed of various chemical components, enabling it to exert multiple pharmacological effects. However, the majority of current research primarily focuses on the impact of its main chemical constituents. Traditional Chinese medicine emphasizes the holistic concept and the systemic in disease treatment. Consequently, studying DR only through a limited number of chemical components may not adequately capture its full pharmacological potential. Based on the literature we have reviewed, most existing clinical trials involving DR are characterized by small sample sizes, which may not accurately represent their true clinical efficacy. For example, in the study conducted by Bao et al. (2015), the sample size was limited to 80 participants, which may introduce several biases, including increased sampling error, selection bias, reduced accuracy, and challenges in detecting small effects. To mitigate these biases, researchers should strive to select a sufficiently large sample size and ensure that the sample is representative of the population. At the same time, employing appropriate statistical methods and models is crucial for correcting and minimizing bias. It is also important to select patients strictly during the patient recruitment phase. It is crucial to acknowledge that few of the clinical studies have conducted long-term follow-ups with patients to evaluate the sustained efficacy of DR treatment. Cardiovascular disease is typically chronic and progressive, patient conditions can evolve with time. Longitudinal follow-up allows for dynamic monitoring of disease progression, thereby capturing changes on time. Analysis of the collected data can provide deeper insights into the persistence of DR treatment effects and identify any potential delayed adverse reactions, thus offering more scientifically sound and clinically relevant guidance. Therefore, it needs to conduct large-scale, high-quality randomized controlled trials in the future to assess the efficacy and safety of DR in patients with various cardiovascular diseases. Additionally, elucidating the pharmacological effects of all components in DR is of positive significance for cardiovascular disease treatment. This approach will help provide a theoretical foundation for its clinical applications and highlight its promising prospects in the cardiovascular field. In conclusion, DR exhibits significant potential for application in the treatment of cardiovascular diseases owing to its chemical constituents and biological activities. With the development of nanotechnology, drug delivery based on cardiac-targeted nanocarriers has become an effective method of treating heart-related diseases (Zhang et al., 2023; Soumya and Raghu, 2023). Combining DR with nanoparticles and nanocarriers may allow it to target cardiac tissue with higher target specificity and sensitivity, thereby improving therapeutic efficacy. Numerous emerging technologies and material innovations also offer new opportunities for treating cardiac diseases, including drug coatings (Hu et al., 2023). This approach may provide direction for the future development of DR in treating cardiovascular diseases. Nevertheless, further research is essential to fully realize this potential. DR may ultimately offer a novel approach to managing cardiovascular diseases, thereby providing safer and more effective treatment options for patients.

## Abbreviations

CaMK, calcium/calmodulin-dependent protein kinase; CLC, compound longxuejie capsule; CHD, coronary atherosclerotic heart disease; CK, creatine kinase; CK-MB, creatine kinase MB; DR, *Draconis Resina*; HMGB1, high mobility group box 1 protein; HFD, high-fat diet; HASMC, human aortic smooth muscle cells; ICAM-1, intercellular adhesion molecule-1; I/R, ischemia/reperfusion; LDH, lactate dehydrogenase; LPS, lipopolysaccharide; LTC, longxue tongluo capsule; MDA, malondialdehyde; MCP-1, monocyte chemotactic protein-1; MIRI, myocardial ischemia-reperfusion injury; NF-κB, nuclear factor kappa B; PAI-1, plasminogen activator inhibitor-1; PDGF, platelet-derived growth factor; ROS, reactive oxygen species; SOD, superoxide dismutase; tPA, tissue-type plasminogen activator; TGF-β1, transforming growth factor-β1; uPA, urokinase-type plasminogen activator; VCAM-1, vascular cell adhesion molecule-1; VEGFA, vascular endothelial growth factor.
